# Supplementary material for: Feedback regulation of heat shock factor 1 (Hsf1) activity by Hsp70‐mediated trimer unzipping and dissociation from DNA
Source: EMBO J. 2020 Jun 3;39(14):e104096. doi: 10.15252/embj.2019104096 (PMC7360973; doi:10.15252/embj.2019104096)
Supplement: Supplementary file 1 — Appendix [file EMBJ-39-e104096-s001.pdf]

Hsp70s dissociate Hsf1 trimers from DNA by unzipping the trimerization domain

Szymon W. Kmiecik, Laura Le Breton, and Matthias P. Mayer

Appendix: Supplemental Information, Figures and Tables

Table of Contents

Supplemental Information 1
Equation for fitting the dissociation data of mixtures of Hsf1wt and Hsf1Δ(202-213).......... 1
Supplemental Figures 3
Supplemental Tables 10

Supplemental Information

Equation for fitting the dissociation data of mixtures of Hsf1wt and Hsf1Δ(202-213).

When mixing Hsf1wt and Hsf1Δ(202-213), homo- and heterotrimers will form according to a binomial distribution $(na + mb)^3$ with n/m being the Hsf1wt to Hsf1Δ(202-213) ratio.

Assuming that a single Hsc70 binding site is sufficient for Hsf1 dissociation and that the number of binding sites does not influence the rate of dissociation the following equation would describe the reaction:

$$If\ t < t_0\ then\ y = y_{max}$$

$$ELSE\ y = y_{0,wt} + (y_{max} - y_0) * f_\Delta + (y_{max} - y_0) * (1 - f_\Delta) * e^{-k(t-t_0)}$$

with y_{max} and y_0 representing the fitted maximal and minimal fluorescence polarization values for wild type Hsf1, k being the rate of the dissociation reaction, and f_Δ being the fraction of Hsf1Δ(202-213) homotrimers, which cannot be dissociated. Simulated results of this equation are shown in Fig EV2A as dashed lines.

Assuming that the number of HR-B proximal Hsc70 binding sites available in the Hsf1 trimer influences the rate by which Hsf1 timers are dissociated, the following equation system of a sum of three exponential functions weighted by the relative abundance of the individual species has to be used:

$For\ Hsf1wt{:}\ Hsf1\Delta(202-213) = 3{:}0$

$$If\ t < t_0\ then\ y = y_{max}$$

$$ELSE\ y = y_0 + (y_{max} - y_0) * e^{-k_3(t-t_0)}$$

$For\ Hsf1wt{:}\ Hsf1\Delta(202-213) = 2{:}1$

$$If\ t < t_0\ then\ y = y_{max}$$

$$ELSE\ y = y_0 + (y_{max} - y_0) * \left(\frac{6}{27}e^{-k_1(t-t_0)} + \frac{12}{27}e^{-k_2(t-t_0)} + \frac{8}{27}e^{-k_3(t-t_0)}\right)$$

$For\ Hsf1wt{:}\ Hsf1\Delta(202-213) = 1{:}1$

$$If\ t < t_0\ then\ y = y_{max}$$

$$ELSE\ y = y_0 + (y_{max} - y_0) * \left(\frac{3}{8}e^{-k_1(t-t_0)} + \frac{3}{8}e^{-k_2(t-t_0)} + \frac{1}{8}e^{-k_3(t-t_0)}\right)$$

$For\ Hsf1wt{:}\ Hsf1\Delta(202-213) = 1{:}2$

$$If\ t < t_0\ then\ y = y_{max}$$

$$ELSE\ y = y_0 + (y_{max} - y_0) * \left(\frac{12}{27}e^{-k_1(t-t_0)} + \frac{6}{27}e^{-k_2(t-t_0)} + \frac{1}{27}e^{-k_3(t-t_0)}\right)$$

with y_{max} and y_0 representing the fitted maximal and minimal fluorescence polarization values and k_1, k_2, k_3 being the rates of the dissociation reaction if 1, 2 or 3 Hsc70 binding sites are available per Hsf1 trimer. This equation system was fitted globally to the data of Fig 5A. The results are shown in Fig EV2C as solid lines. Fig EV2D shows the resulting rates (mean ± SD; n = 4).

Supplemental Figures

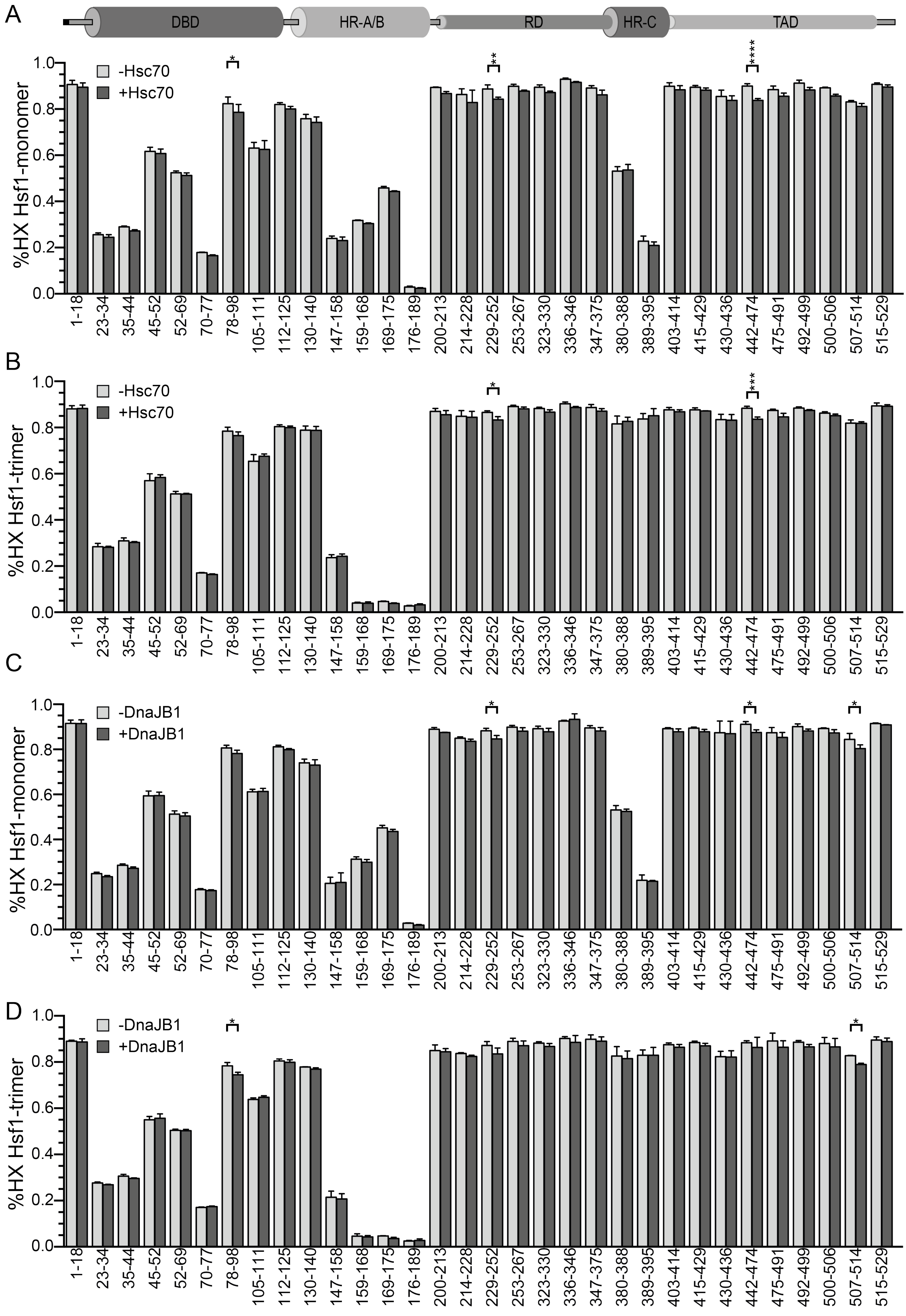

Appendix Figure S1: Hsc70 and DnaJB1 protect specific regions in Hsf1 monomers and trimers. Relative deuteron incorporation rates in monomeric (**A**, **C**) and trimeric (**B**, **D**) Hsf1 in the absence (light gray bars) or presence of Hsc70 (A, B; dark gray bars) or DnaJB1 (C, D; dark gray bars). Values represent means ± SD (n = 3); *, $p \leq 0.05$; **, $p \leq 0.01$; ***, $P \leq 0.001$; ANOVA, Sidak's multiple comparison test.

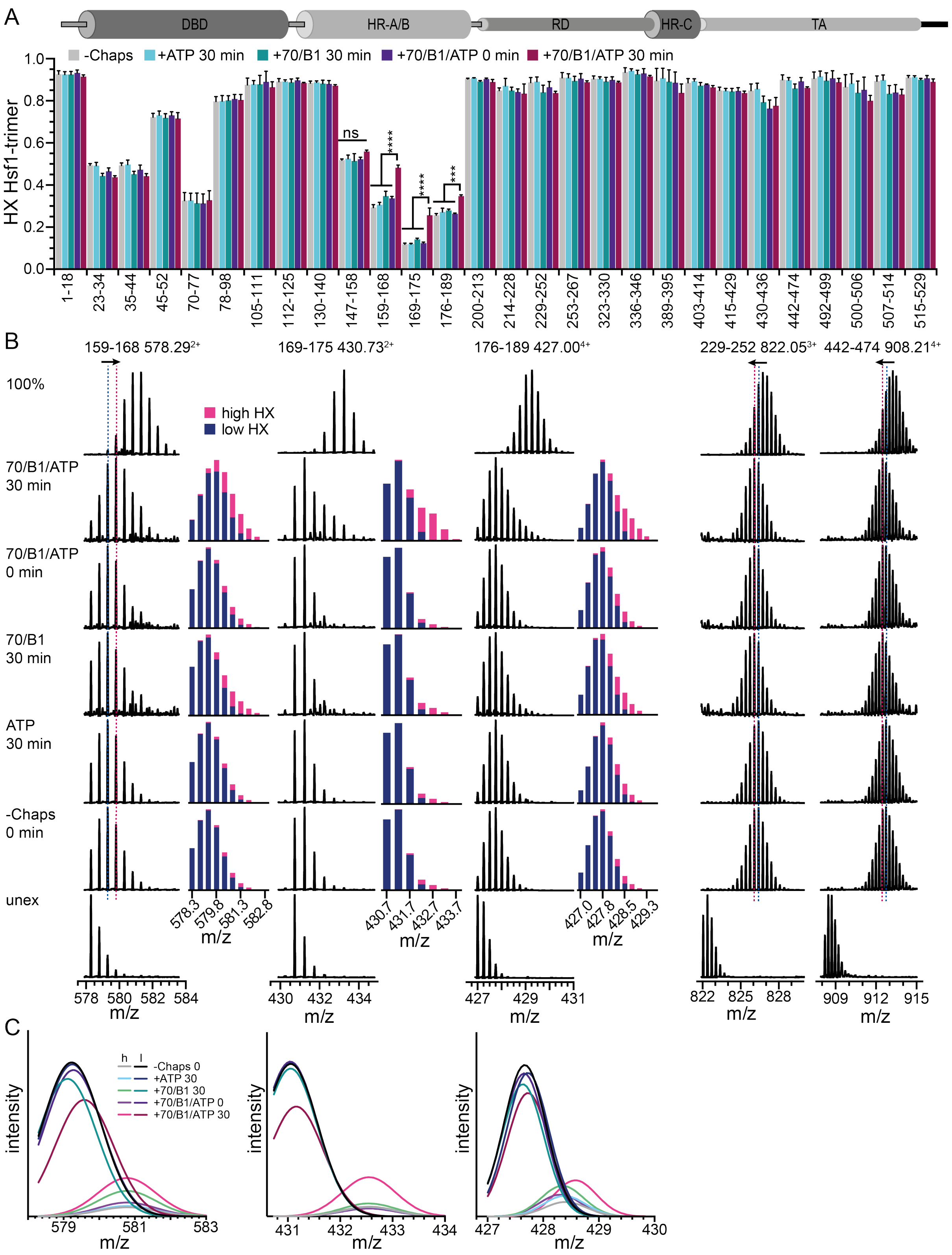

Appendix Figure S2: Hsc70 and DnaJB1 induce local unfolding in the trimerization domain of Hsf1.

A Fractional hydrogen exchange in Hsf1 segments as indicated upon incubation for 300 s at 25°C in D_2O buffer in the absence of chaperones (-Chaps), or after a preincubation of

trimeric Hsf1 in the presence of ATP, Hsc70 (70) and DnaJB1 (B1) for 0 or 30 min as indicated. Values represent means ± SD (n = 3). Statistical significance was established by ANOVA with Sidak's multiple comparison (ns, not significant; ***, $p \leq 0.001$; ****, $p \leq 0.0001$). On top, cartoon of Hsf1 domains to locate the exchanging segments.

B Original spectra of peptic peptides 578.29^{3+} (amino acids 159-168), 430.73^{2+} (aa 169-175), 427.00^{4+} (aa 176-189), 822.05^{3+} (aa 229-252), and 908.21^{4+} (aa 442-474) from unexchanged Hsf1 (unex), the 100% deuterated Hsf1 (100%), and Hsf1 incubated at 25°C for 300 s in D_2O buffer in the absence of Hsc70, DnaJB1 and ATP (-Chaps) or after preincubation in the presence of Hsc70 (70), DnaJB1 (B1) and ATP for 0 or 30 min as indicated. The bar graphs to the right of the spectra show the fractional peak intensities of the low (dark blue) and high (magenta) exchanging subpopulation as calculated from the fit of two Gaussian distributions to the maximal isotope peak intensities (panel C and Fig 3C). Blue and red dashed lines in spectra for peptides 578.29^{3+}, 822.05^{3+}, and 908.21^{4+} indicate the highest peak in the samples without chaperones and the sample with Hsc70, DnaJB1 and ATP incubated for 30 min, respectively. Arrows on top of the dashed lines indicate the Hsc70-mediated overall change in deuteron incorporation.

C Individual Gaussian distributions for the high (h) and low (l) exchanging species for the different conditions the sum of which results in the curves in Fig 3C.

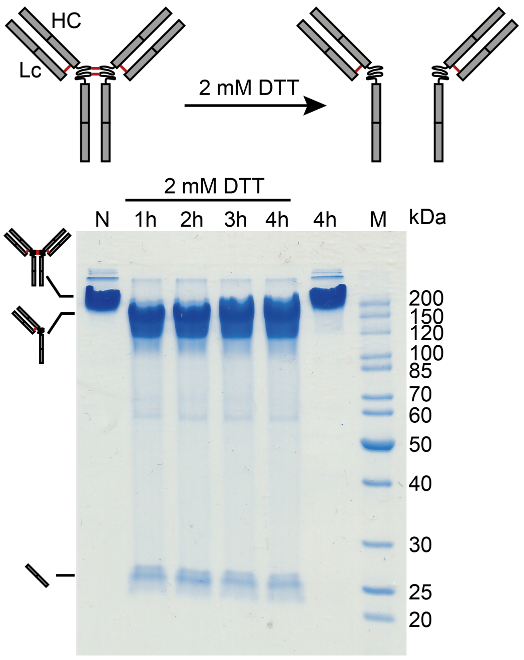

Appendix Figure S3: Anti-FLAG antibodies are split in halfmers by incubation with DTT. Samples were incubated in the absence (N and 4h) or in the presence of 2 mM DTT for 1 to 4 h, as indicated, separated by non-reducing SDS-polyacylamide gel electrophoresis, and the gel subsequently stained by Coomassie Brilliant Blue. M, protein size marker.

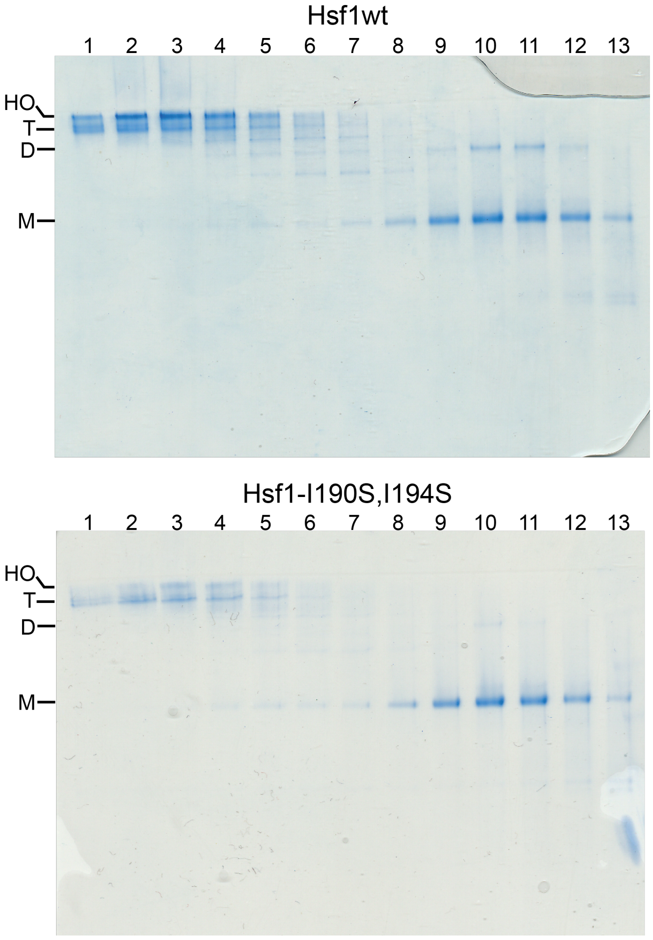

Appendix Figure S4: Hsf1-I190S,I194S forms less trimers and higher order oligomers than Hsf1wt. Monomeric and trimeric species of Hsf1wt (upper panel) and Hsf1-I190S,I194S (lower panel) freshly purified from an overproducing *E. coli* strain were separated by size exclusion chromatography and fractions were analyzed by BN-PAGE. Lanes 1-13 consecutive fractions from size exclusion chromatography. HO, higher order oligomers; T, Hsf1 trimers; D, Hsf1 dimers; M, Hsf1 monomers.

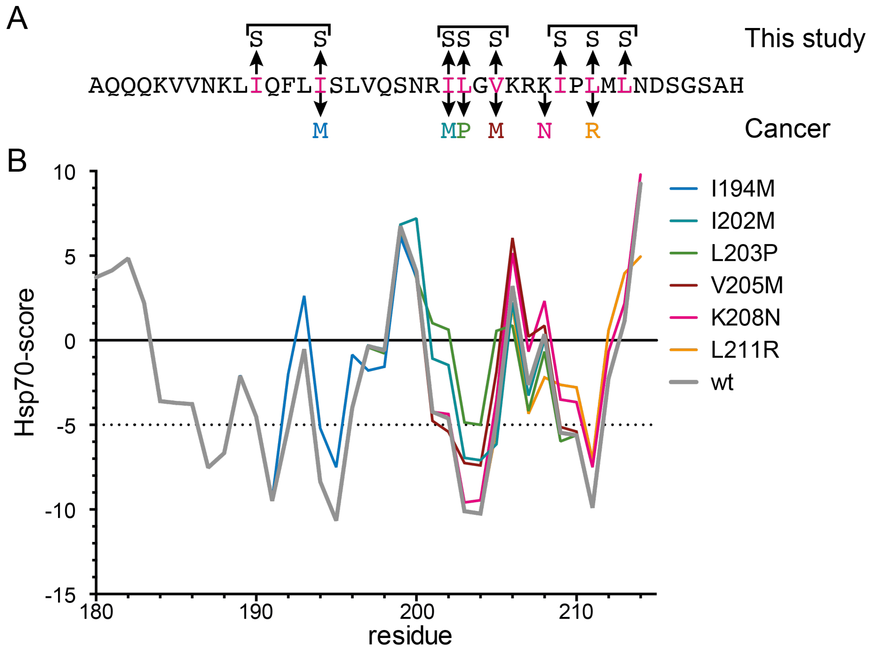

Appendix Figure S5: Cancer genomes reveal mutations in Hsf1 that would be expected to affect Hsc70/Hsp70-mediated monomerization of Hsf1.

A Hsf1 sequence from 180 to 215 with hydrophobic residues replaced by serine in our study in magenta (brackets indicate multiple mutations within the same Hsf1 variant). Below the sequence mutations found in cancer genomes (COSMIC v.90, 5.9.2019; https://cancer.sanger.ac.uk/cosmic).

B Hsp70 score calculated by the Hsp70 binding site algorithm for Hsf1 residues 180 to 215 with single residues replaced as indicated. Values below the dashed line (-5) predict sites that are likely to be bound by Hsp70.

Supplemental Tables

Appendix Table S1: Species selected for multiple sequence alignments

CODE	Species name	Taxonomy	Accession number
**Metazoa**			
**HUMAN**	*Homo sapiens*	Metazoa, Vertebrata, Mammalia	HSF1_HUMAN Q00613
**CHICK**	*Gallus gallus*	Metazoa, Vertebrata, Aves	HSF1_CHICK P38529
**ANOSA**	*Anolis sagrei*	Metazoa, Vertebrata, Sauropsida	A0A218PFT7_ANOSA
**XENLA**	*Xenopus laevis*	Metazoa, Vertebrata, Amphibia	HSF_XENLA P41154
**LATCH**	*Latimeria chalumnae*	Metazoa, Vertebrata, Coelacanthimorpha	H3AZK4_LATCH
**DANRE**	*Danio rerio*	Metazoa, Vertebrata, Teleostei	Q9IAS0_DANRE
**CALMI**	*Callorhinchus milii*	Metazoa, Vertebrata, Chondrichthyes	V9KGH9_CALMI
**STIJA**	*Stichopus japonicus*	Metazoa, Echinodermata, Holothuroidea	A0A2G8KH32_STIJA
**UREUN**	*Urechis unicinctus*	Metazoa, Annelida, Polychaeta	W8PKQ0_UREUN
**ELYCH**	*Elysia chlorotica*	Metazoa, Mollusca, Gastropoda	A0A433SUE2_ELYCH
**CRAGI**	*Crassostrea gigas*	Metazoa, Mollusca, Bivalvia	F8WKQ0_CRAGI
**DAPPU**	*Daphnia pulex*	Metazoa, Arthropoda, Crustacea	E9GSC3_DAPPU
**STRMM**	*Strigamia maritima*	Metazoa, Arthropoda, Myriapoda	T1J4W2_STRMM
**DROME**	*Drosophila melanogaster*	Metazoa, Arthropoda, Insecta	HSF_DROME P22813
**AMBMA**	*Amblyomma maculatum*	Metazoa, Arthropoda, Chelicerata	G3MIG2_9ACAR
**CAEEL**	*Caenorhabditis elegans*	Metazoa, Nematoda	G5EFT5_CAEEL
Fungi			
**YEAST**	*Saccharomyces cerevisiae*	Fungi, Dikarya, Ascomycota, Saccharomycotina	HSF_YEAST P10961
**PODCO**	*Podospora comata*	Fungi, Dikarya, Ascomycota, Pezizomycotina	A0A447C618_9PEZI
**ASPFU**	*Aspergillus fumigatus*	Fungi, Dikarya, Ascomycota, Pezizomycotina	Q4WE63_ASPFU
**SCHPO**	*Schizosaccharomyces pombe*	Fungi, Dikarya, Ascomycota, Taphrinomycotina	HSF_SCHPO Q02953
**TAPDE**	*Taphrina deformans*	Fungi, Dikarya, Ascomycota, Taphrinomycotina	R4XB54_TAPDE
**SPIPD**	*Spizellomyces punctatus*	Fungi, Fungi incertae sedis, Chytridiomycota	A0A0L0H754_SPIPD
**CONCO**	*Conidiobolus coronatus*	Fungi, Fungi incertae sedis, Zoopagomycota	A0A137P9M3_CONC2
**MOREL**	*Mortierella elongata*	Fungi, Fungi incertae sedis, Mucoromycota	A0A197KA09_9FUNG
