# Supplementary material for: Feedback regulation of heat shock factor 1 (Hsf1) activity by Hsp70‐mediated trimer unzipping and dissociation from DNA
Source: EMBO J. 2020 Jun 3;39(14):e104096. doi: 10.15252/embj.2019104096 (PMC7360973; doi:10.15252/embj.2019104096)
Supplement: Supplementary file 2 — Expanded View Figures PDF [file EMBJ-39-e104096-s002.pdf]

Expanded View Figures

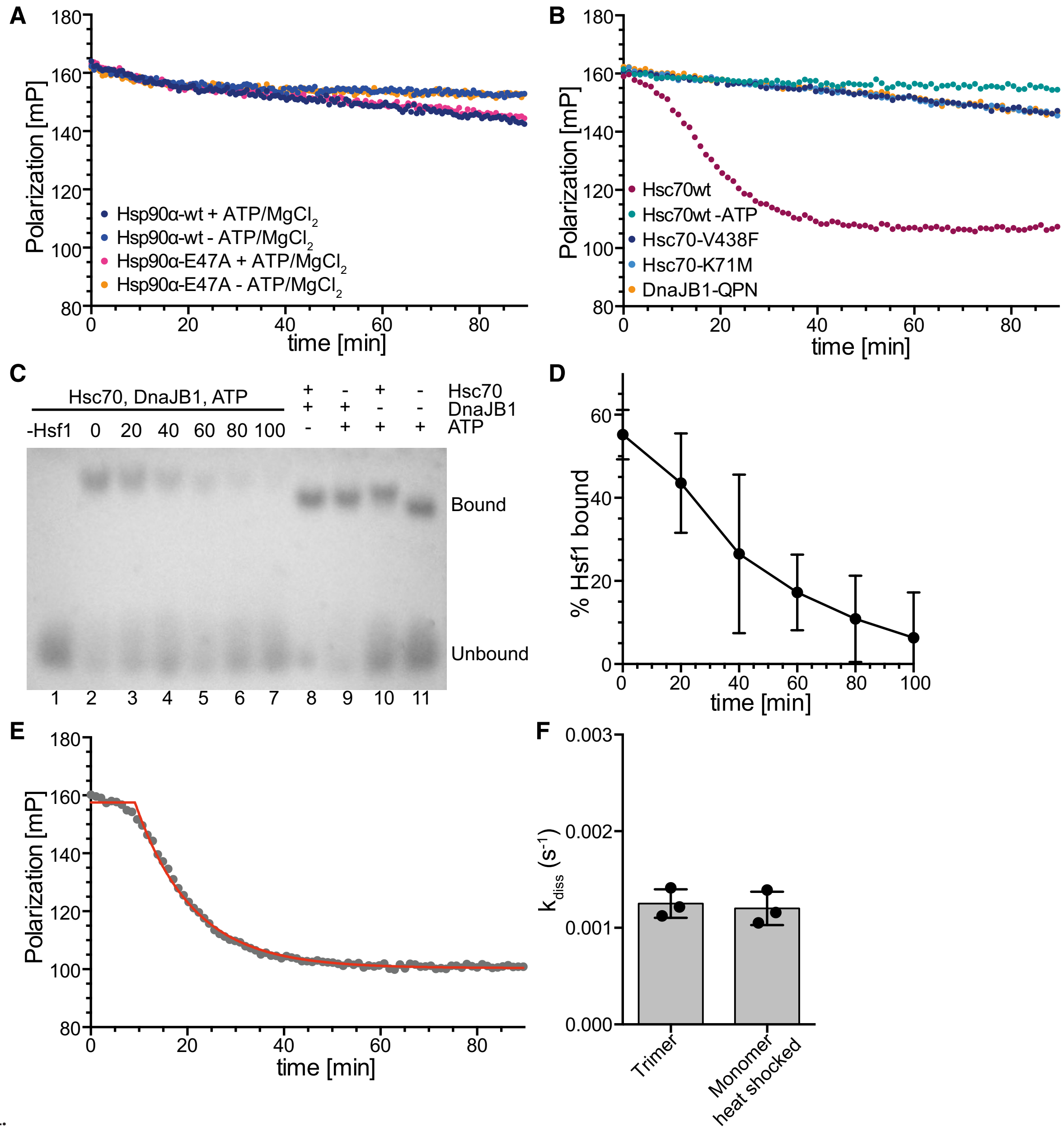

Figure EV1.

Figure EV1. Hsc70 dissociates Hsf1 from DNA.

A Hsp90α wild type or ATP hydrolysis deficient Hsp90α-E47A do not influence Hsf1 DNA binding. Trimeric Hsf1 was bound to Alexa Fluor® 488-labeled HSE-DNA and the indicated components added at timepoint 0. A representative experiment is shown.

B Hsc70's ability to hydrolyze ATP and to bind to polypeptides, and stimulation of Hsc70's ATPase activity by DnaJB1 are essential for dissociation of DNA-bound Hsf1. Hsc70-K71M, ATPase deficient; Hsc70-V438F, reduced affinity for polypeptides; DnaJB1-H32Q,D34N (DnaJB1-QPN), defective in stimulation of Hsc70's ATPase activity.

C Hsc70/DnaJB1-mediated dissociation of Hsf1 from HSE-DNA analyzed by electrophoretic mobility shift assay. Trimeric Hsf1 was bound to Cy3-labeled HSE-DNA and the indicated components added at timepoint 0. The reaction mixture was separated on a 1% agarose gel. Lane 1, Cy3-labeled HSE-DNA without Hsf1 in the presence of Hsc70, DnaJB1, and ATP; lanes 2 to 7; dissociation reaction after 0- to 100-min incubation; and lanes 8 to 11, dissociation reaction with missing components after 100-min incubation.

D Quantification of EMSA shown in (C) and two similar EMSAs. Shown are mean ± SD (n = 3).

E Non-linear regression analysis of the representative data shown in Fig 1D (magenta dots, shown here in gray). The red line represents the fit of the composite equation $y = y_{max}$ for $t \leq t_0$ and $y = y_0 + (y_{max} - y_0) * e^{-k(t-t_0)}$ for $t > t_0$ with y_{max} and y_0 signifying the fitted maximal and minimal fluorescence polarization values and k being the rate of the dissociation reaction, to these exemplary data.

F The dissociation rate did not differ, if Hsf1 purified as a trimer from *E. coli* without prior heat shock, or monomeric Hsf1 heat shocked for 10 min at 42°C, was used for the reaction. Shown are mean ± SD (n = 3).

Source data are available online for this figure.

 © 2020 The Authors

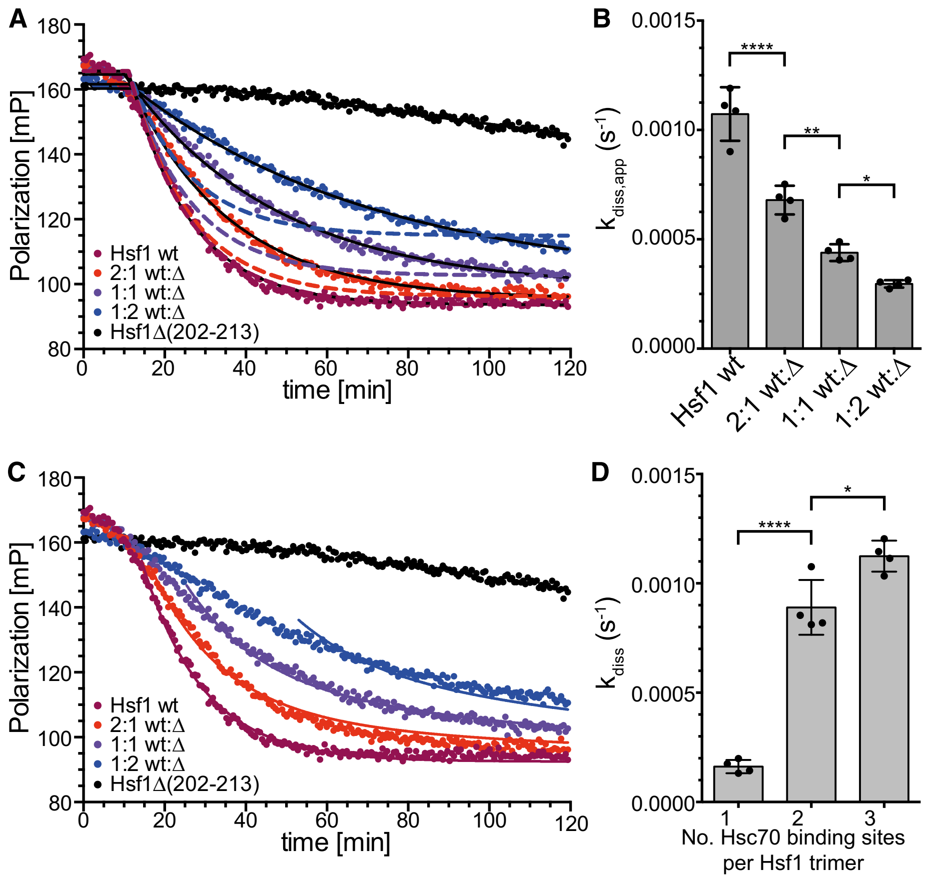

Figure EV2. The rate of Hsc70/DnaJB1-mediated dissociation of Hsf1 from HSE-DNA depends on the number of HR-B proximal Hsc70 binding sites.

A Non-linear regression analysis of the data from Fig 5A using the composite exponential decay function shown in Fig EV1E yielding the rates of the dissociation reaction shown in panel (B) for all independent experiments performed. The solid black lines represent the fits of this decay function to the individual data points. Dashed lines represent a theoretical dissociation function assuming that a single Hsc70 binding site is sufficient for Hsf1 trimer dissociation with maximal rate.

B Hsf1 dissociation rates for the different Hsf1wt:Hsf1Δ(202–213) mixtures fitted with the function from Fig EV1E. Shown are mean ± SD (n = 4); ANOVA, Sidak's multiple comparison; *P < 0.05; **P < 0.01; ****P < 0.0001.

C Non-linear regression analysis of the data from Fig 5A using a global fit of the function shown in Appendix that assumes that the number of HR-B proximal Hsc70 binding sites influences the rate of dissociation of Hsf1 bound to HSE-DNA. The solid lines represent the fit of this function yielding the different dissociation rates k_1, k_2, and k_3 for the dissociation reaction of Hsf1 with one, two, or three HR-B proximal Hsc70 binding sites per Hsf1 trimer shown in panel (D) for all independent experiments performed.

D Dissociation rates derived from the global fit of panel (C) for Hsf1 trimers with a single (k_1), two (k_2), or three (k_3) HR-B proximal Hsc70 binding sites available; mean ± SD (n = 4); ANOVA, Sidak's multiple comparison; *P < 0.05; ****P < 0.0001.

© 2020 The Authors

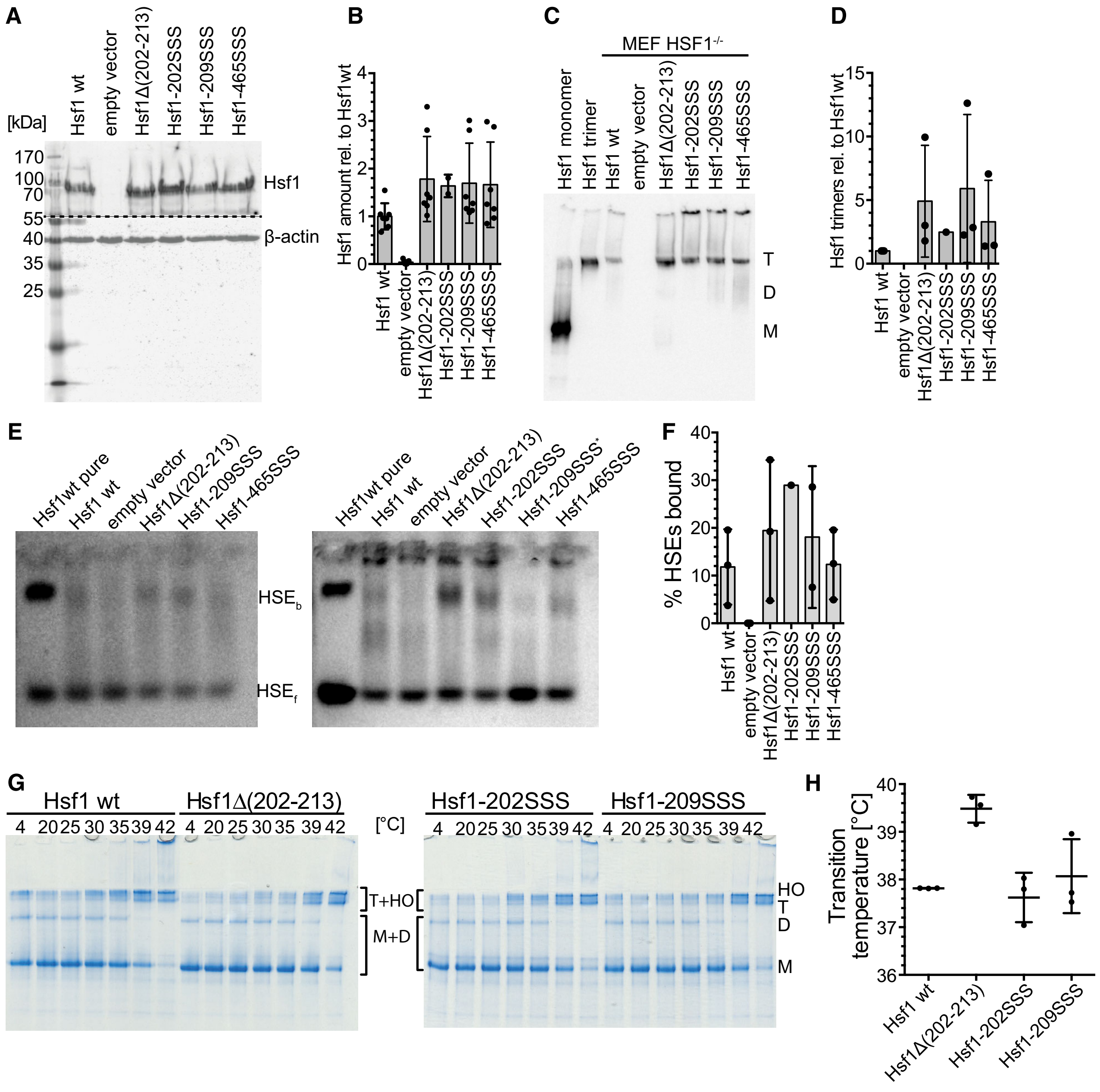

Figure EV3.

 © 2020 The Authors

Figure EV3. Wild-type Hsf1 and mutant Hsf1 are expressed to the same levels in transfected HSF1$^{-/-}$ MEFs and form trimers in cultured cells that are able to bind to DNA.

A Cell lysates of HSF1$^{-/-}$ MEFs transfected with constructs encoding the indicated proteins were separated by SDS–PAGE and blotted onto PVDF membranes, and Hsf1 and β-actin were detected using specific antisera. Before the immune detection, the blot was cut at the dashed line. Representative blot of three independent transfection experiments is shown.

B Quantification of the blot shown in (A) and similar blots. No significant differences between wild-type and mutant proteins were detected (ANOVA, Sidak's multiple comparison). Shown are mean ± SD (n = 7, except for Hsf1-202SSS: n = 2).

C Cell lysates of HSF1$^{-/-}$ MEFs transfected with constructs encoding the indicated proteins were separated by BN-PAGE and blotted onto PVDF membranes, and Hsf1 was detected using specific antisera. As a control, purified monomeric and trimeric Hsf1 was loaded in lanes 1 and 2. M, monomer. D, dimer. T, trimer.

D Quantification of blot shown in (C) and similar blots. Shown are mean ± SD (n = 3, except for Hsf1-202SSS, for which a single experiment is shown)

E Electrophoretic mobility shift assay detects DNA binding of Hsf1 in cell lysate. Cell lysates of HSF1$^{-/-}$ MEFs transfected with constructs encoding the indicated proteins were incubated with Cy3-labeled HSE-DNA and separated on 1% agarose gels. Hsf1 bound (HSE_b) and free (HSE_f) DNA were detected by fluorescence scanning. EMSAs of two of three independent transfection experiments are shown. *, due to increased cell death, lysate of MEFs transfected with Hsf1-209SSS encoding plasmid was not concentrated enough, and HSE-DNA was only incubated with less than half the protein concentration as compared to the other samples; this lane was therefore not quantified.

F Quantification of EMSAs shown in (E) and additional ones. Mean ± SD are shown (n = 3, except for Hsf1-209SSS: n = 2 and Hsf1-202SSS: n = 1).

G Hsf1 variants with mutations in the HR-B proximal Hsc70 binding site do not trimerize at lower transition temperatures. Purified monomeric Hsf1 wild-type and mutant proteins were incubated for 10 min at 4–42°C as indicated and subsequently separated by BN-PAGE.

H Monomer (M), dimer (D), trimer (T), and higher order oligomer (HO) bands of three independent experiments were quantified, and the equation for the thermal unfolding transition fitted to the data. The graph shows the melting temperature ± SD (n = 3).

Source data are available online for this figure.

© 2020 The Authors

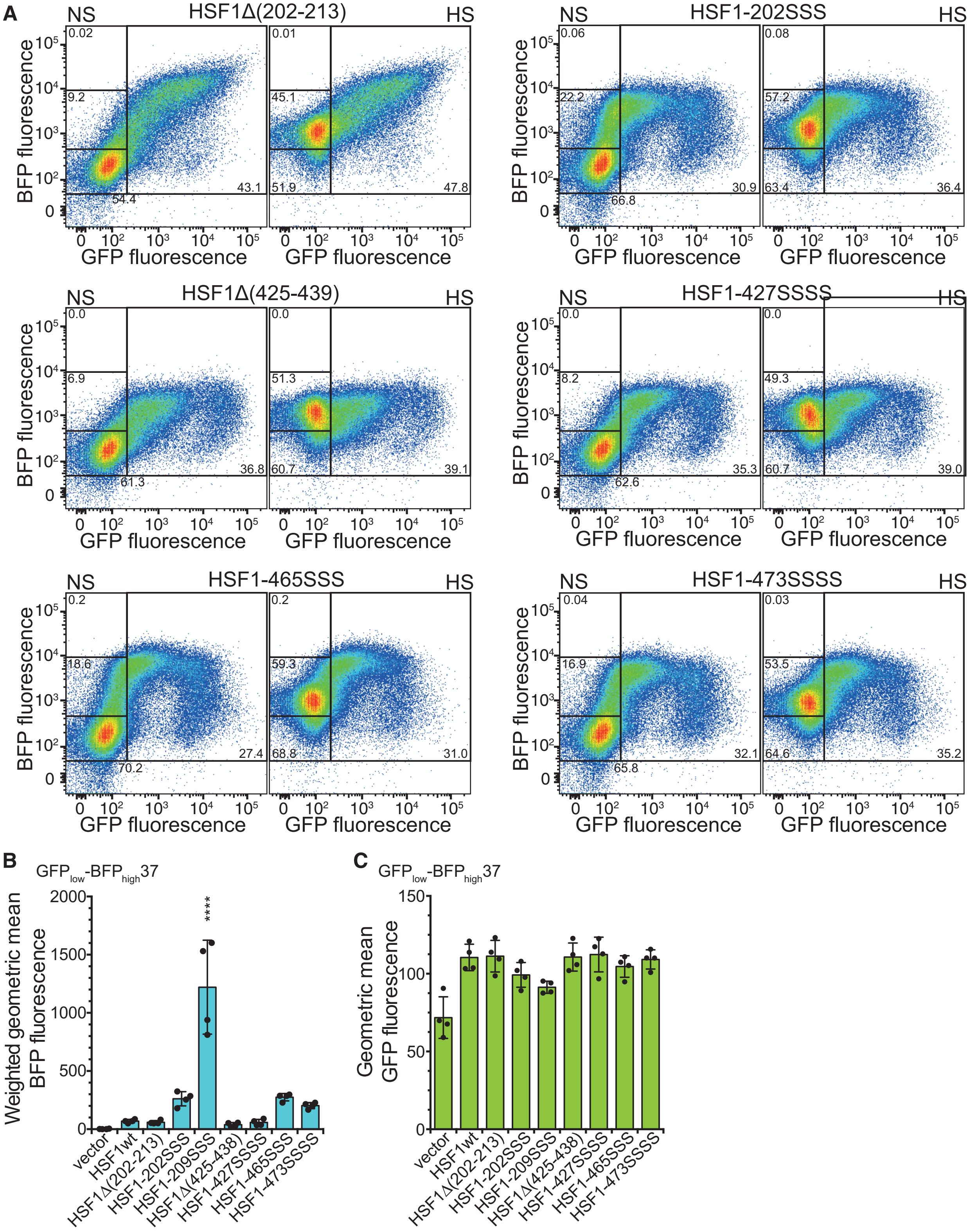

Figure EV4.

 © 2020 The Authors

Figure EV4. Compromising Hsc70 binding potentiates expression of a heat shock reporter under non-stress and heat shock conditions in HeLa cells.

A Exemplary flow cytometry data plotting BFP fluorescence versus GFP fluorescence, using the same gates for analysis as shown in Fig 7B. Numbers indicate the fraction (%) of cells (forward scatter singlets) in each gate (number of GFP_{low} is underneath the gate for NS data). Color indicates the cell density from dark blue (low) to red (high). NS, cells grown at 37°C; HS, cells heat shocked at 43°C for 1 h and recovered for 3 h at 37°C.

B Geometric mean of BFP fluorescence weighted by the relative number of cells in the GFP_{low}-BFP_{high}37 gate (fluorescence * fraction forward scatter singlets). Note that the GFP_{low}-BFP_{high}37 gate also comprises the GFP_{low}-BFP_{high}43 gate. Geometric mean ± SD of four independent experiments; ANOVA Sidak's multiple comparison, ****$P < 0.0001$.

C Geometric mean of GFP fluorescence in the GFP_{low}-BFP_{high}37 gate. Hsf1-202SSS, Hsf1-I202S,L203S,V205S; Hsf1-209SSS, Hsf1-I209S,L211S,L213S; Hsf1-427SSSS, Hsf1-M427S,L429S,L432S,L436S; Hsf1-465SSS, Hsf1-L465S,V466S,Y468S; Hsf1-473SSSS, Hsf1-L473S,F474S,L475S,L476S; geometric mean ± SD of 4 independent experiments.

Figure EV5. Hsc70 binding sites important for unzipping the Hsf1 trimer are conserved in metazoa but not in yeast.

A Clustal Ω multiple sequence alignment of representative Hsf1 sequences of metazoan (top) and fungal (bottom) phyla, comprising the trimerization domain (HR-A and HR-B) and the HR-B-proximal region of the regulatory domain, containing the Hsc70 binding sites necessary for Hsc70/DnaJB1-mediated monomerization of trimeric human Hsf1. For species names and phyla, see Appendix Table S1 (below). A number of starting and ending residues are indicated for each sequence. Hydrophobic residues are colored in green, positively charged in blue, and negatively charged in magenta.

B–D Hsp70 binding site prediction algorithm suggests good Hsp70 binding sites for all metazoan and some fungal Hsf1s. Left panels solid lines, average Hsp70 prediction score for vertebrate (B), invertebrate (C), and fungal (D) Hsf1 sequences assigned to the center residue of the 13mer segment used to calculate the score (Rüdiger *et al*, 1997) and numbered according to human Hsf1; black dashed lines, standard deviation of the calculated scores. All values ≤ −5 (red lines) indicate an 80% chance of being a good Hsp70 binding site. Right panels, number of segments of each sequence within the region corresponding to residues 180–200 (gray bars) and 201–214 (black bars) of human Hsf1 with a score ≤ −5.

© 2020 The Authors

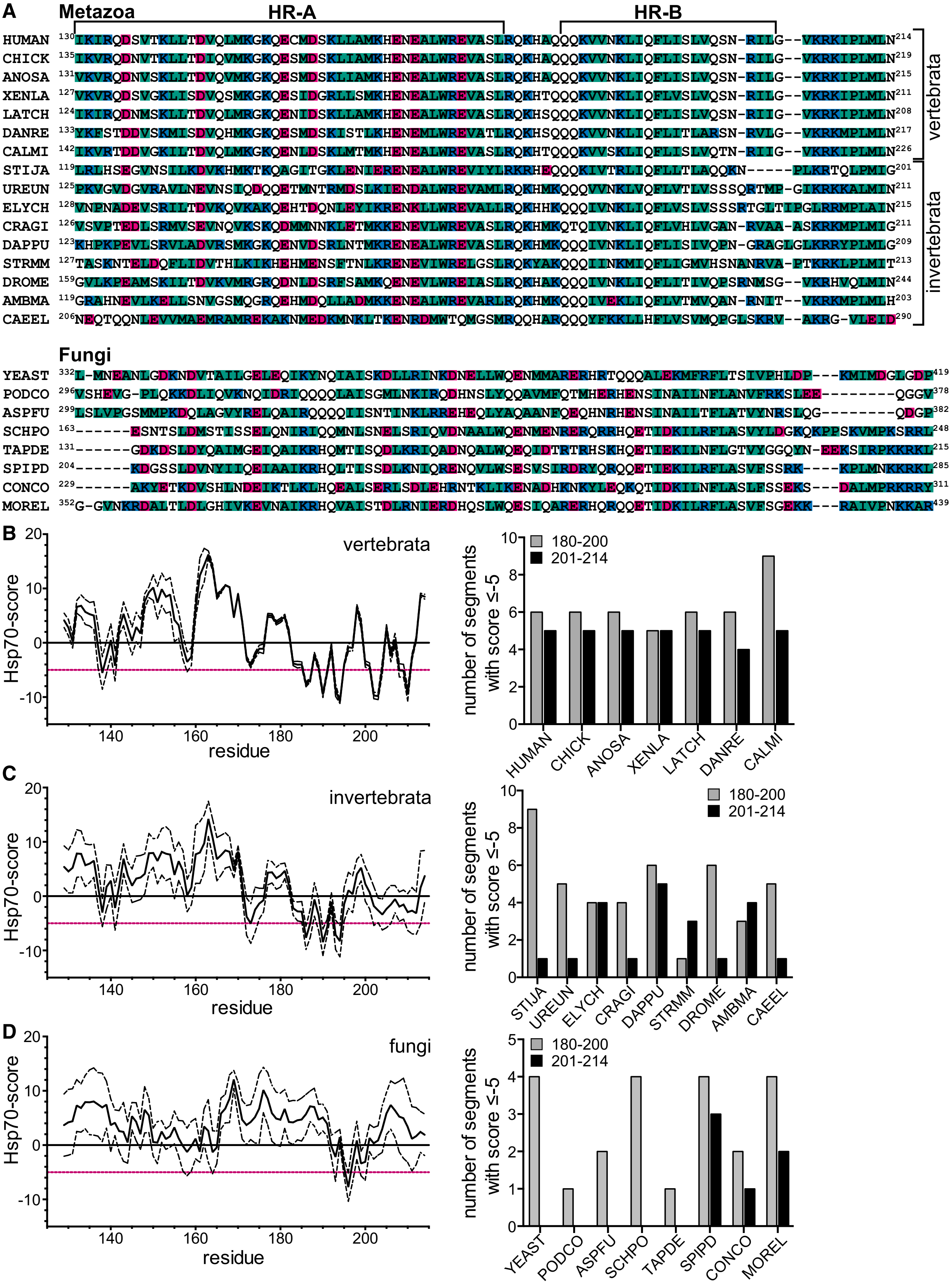

Figure EV5.

© 2020 The Authors
